# The MEDITAGING study: protocol of a two-armed randomized controlled study to compare the effects of the mindfulness-based stress reduction program against a health promotion program in older migrants in Luxembourg

**DOI:** 10.1186/s12889-023-17387-9

**Published:** 2023-12-11

**Authors:** Ana C. Teixeira-Santos, Leandro Gomes, Diana R. Pereira, Fabiana Ribeiro, Anabela Silva-Fernandes, Carine Federspiel, Jean-Paul Steinmetz, Anja K. Leist

**Affiliations:** 1 Department of Social Sciences, Institute for Research on Socio-Economic Inequality, University of Luxembourg, Esch-sur-Alzette, Luxembourg; 2 Interdisciplinary Postgraduate Program in Human Sciences, State University of Amazonas PPGICH/UEA, Manaus, Brazil; 3 NAURBE Group - Cities, Popular Cultures and Heritage, Federal University of Amazonas - Postgraduate Program in Social Anthropology, Manaus, Brazil; 4 Human Cognition Laboratory – CIPsi, School of Psychology, University of Minho, Braga, Portugal; 5 Psychological Neuroscience Laboratory, CIPsi, School of Psychology, University of Minho, Braga, Portugal; 6 Centre for Memory and Mobility, Zitha, Luxembourg

**Keywords:** Migrant health, Older adults, MBSR, Mindfulness-based interventions, Health promotion program

## Abstract

**Background:**

Migration is a phenomenon worldwide, with older migrants, particularly those with fewer socioeconomic resources, having an increased risk of developing adverse cognitive and health outcomes and social isolation. Therefore, it is of utmost importance to validate interventions that promote healthy aging in this population. Previous studies have shown a positive impact of mindfulness based-stress reduction (MBSR) on outcomes such as cognition and sleep. However, only a few studies verified its potential in older adults, especially with vulnerable populations such as migrants. This article presents the protocol of the MEDITAGING study, which is the first to investigate the MBSR effects in migrants aged ≥55 in comparison to a health promotion program.

**Methods:**

MEDITAGING is a two-arm randomized, double-blinded, controlled study, which will include older Portuguese-speaking migrants (*n* = 90). Participants are randomized to the MBSR or a health promotion program. Both interventions are conducted in groups over a total of 8 weeks, incorporating weekly meetings, an additional 4-hour class, and extra at-home tasks. The health promotion program has the same structure as the MBSR but comprises different activities related to dementia prevention, healthy habits, cognitive stimulation, sleeping, nutrition, watercolor painting, and physical activity. The assessment of executive functioning, physiological stress measures, self-reported questionnaires, and qualitative interviews are conducted at baseline, after 8 weeks (post-intervention), and at a follow-up session (from one to 3 months thereafter). Analyzes will be conducted using a modified intention-to-treat approach (all participants with at least 3 days of participation in the group-sessions and one post-intervention observation).

**Discussion:**

This study will test effects of a mindfulness-based intervention against an active control condition in older adult migrants, which few studies have addressed.

**Trial registration:**

ClinicalTrials.gov NCT05615337 (date of registration: 27 September 2022; date of record verification: 14 November 2022).

**Supplementary Information:**

The online version contains supplementary material available at 10.1186/s12889-023-17387-9.

## Background

Older adults, making up a growing part of aging populations all over the world, have an increased risk of chronic metabolic disorders, cognitive impairment, and dementia [1–3]. Additionally, migration is a phenomenon worldwide, with older migrants, particularly those with less socioeconomic resources, having an increased risk of developing adverse cognitive and health outcomes and social isolation, which may impact the society and increase the need of care to promote health in this population [4, 5]. The Grand Duchy of Luxembourg has an opportune scenario to study this phenomenon, since the large inflow of first-generation immigrants from the 1960s/70s, especially those from Portugal, are currently entering advanced ages. According to the Census data in 2020, more than 47% of the population in the country are foreign-born individuals, from which 34% are made up of Portuguese speakers, with more than 4% of the total population being composed of Portuguese migrants aged 65 and older [6, 7]. Most of these people have very low educational levels (87% of the Portuguese aged between 55 and 64 years old and 80% of those older than 65 have only primary or lower secondary education), occupy low levels of the social structure, and they have poor emotional support, with many facing language barriers as they are unable to speak one of the national languages (i.e., French, German, or Luxembourgish) [7, 8]. These circumstances have a considerable impact in their lives, including an increased risk of dementia [9, 10] and interventions to address this risk are needed for this population.

Mindfulness is the act of intentionally paying attention to the experiences of the present moment, acknowledging thoughts and feelings, and letting them come and go with stillness, without judgment or emotional attachment [11]. Mindfulness training teaches individuals to be attentive, patient, and open to accept positive and negative experiences [12]. This training focuses on sustained and selective attention, as well as cognitive flexibility, which is the ability to adapt to new situations [13, 14]. From a cognitive perspective, the most promising results from mindfulness interventions are found on executive functions such as attention and working memory [15–20]. As the executive functions are diminishing across aging [21] and are transversal to almost all daily activities, restoration or maintenance of these capabilities may amplify the benefits of mindfulness, including a generalization of mindfulness to other domains, as a far transfer effect [22, 23]. The mindfulness-based interventions have also shown considerable benefits on other outcomes such as emotion, depression, stress, anxiety, sleep, quality of life, and mindfulness-related measures [15, 17, 24–27]. However, in general, the mean age of participants in the studies is relatively low [28–33] and some evidence is mixed [34]. The feasibility of a mindfulness-based program has been demonstrated on low-income African American older adults and its effects on blood pressure [35]. The current article describes the study protocol to address the paucity of evidence on effects of mindfulness interventions for older and underserved populations especially with regard to cognitive outcomes.

Beyond effects on cognition, sleep, and mental health, testing possible pathways of an MBRS intervention is advised. Aging is associated with structural and functional loss, underlying the reduction of several cognitive skills, such as attention [36], which when associated with stress, may exacerbate age-related cognitive and physical impairment [37], especially in the most vulnerable populations. Stress may affect neuroendocrine functioning via the hypothalamic-pituitary-adrenal (HPA) axis, causing an exaggerated physiological response and resulting in excessive cortisol production, which is suggested to lead to negative cellular and synaptic changes in the hippocampus and the prefrontal cortex [38]. It has also been linked to inflammatory activities, which may lead to other adverse health outcomes [39–41] as well as the immune system [42]. Thus, a more holistic intervention, focusing on both attention training and stress relief, such as the mindfulness-based stress reduction (MBSR) program, is a promising tool to counteract those age-related conditions [43].

Additionally, other factors appear to modulate mindfulness-related gains. For instance, it was reported that in women, mindfulness training may have a more substantial impact on emotion, i.e., a greater decrease in negative affectivity than men, as well as a more pronounced increase in their mindfulness skills and self-compassion [44]. The number of attended lessons and home practice is also a factor to consider [45]. Thus, it is important to examine which factors may moderate mindfulness training, as they can be used to tailor interventions to better respond to individual differences and needs.

The current article describes the protocol of the MEDITAGING study whose aim is to quantitatively and qualitatively evaluate and compare the effects of the MBSR in Portuguese-speaking migrants in Luxembourg, immediately after and around two-months post-intervention, in comparison to a health promotion program as an active control condition. MEDITAGING is a monocentric, randomized, double-blinded (assessor and participants blind) controlled superiority clinical trial with two parallel arms (with a 1:1 ratio).

## Methods/design

See the Supplementary Material for the Standard Protocol Items Recommendations for Intervention Trials (SPIRIT) 2013 checklist.

### Study setting

The group sessions are conducted at the premises of Zitha, a middle-sized company providing geriatric and gerontological care and assistance to older people living in Luxembourg, and other collaborating institutions such as senior public services and recreation clubs of Luxembourg. Data is being collected with Portuguese-speaking migrants invited through these institutions and through wide dissemination in the local media.

### Participants

Participants are 55 years older and over, community-dwelling, Portuguese-speaking immigrants, living in Luxembourg or frontier regions. According to our eligibility criteria (see Table 1), participants have no major neurological or psychiatric disorders or current unstable illness. Moreover, as we previously verified that Portuguese older adults often present symptoms of anxiety, depression, pain, and sleep disorders [46, 47], we opted for broader inclusion criteria to maximize ecological validity. Additionally, we believe that participants presenting these symptoms would most likely benefit from the interventions [48]. Therefore, we did not exclude participants with corresponding symptoms. The decision to use the age of participants starting at 55 was supported by the evidence that much of the cognitive decline observed in old adults already starts at middle age [49], so we expected that individuals from this age group could benefit from our interventions to preserve cognitive functioning. Additionally, we only included Portuguese-speaking participants as they represent the largest (more than a third) and most vulnerable group of immigrants in Luxembourg [7].

**Table 1:** Eligibility criteria

Inclusion	Exclusion
- Being a Portuguese-speaking migrant, residing in the Grand-Duchy of Luxembourg or the Greater Region; - Age: 55 years old or older; - Mastery of spoken, written, and reading Portuguese; - Subjects without cognitive impairment - verified through the MMSE, cut-off of < 22 adopted since most participants are expected to have a low education level (Kochhann et al., 2010). - Full capacity of consent.	- Previous (up to 3 years before) or current weekly participation in meditation, yoga, or mindfulness-based interventions; - Concomitant participation in another kind of group interventions (e.g., cognitive training, psychological therapy); - Attendance in psychological consultation; - Severe hearing or visual impairment (not corrected); - Severe medical condition requiring intensive medical care that makes it difficult to participate in the group sessions. - Refusal to sign the informed consent; - Diagnosis of dementia; - Clinical neurodegenerative illness, psychotic disorder, unstable psychiatric condition which makes it difficult to participate in group work, posttraumatic stress disorder or history of trauma, acute psychosis, mania, suicidality, or substance abuse within the last 6 months (cutoff: 2 points at the Adult DSM-5 Self-Rated Level 1 Cross-Cutting Symptom Measure).

*DSM-5* Diagnostic and Statistical Manual of Mental Disorders, Fifth Edition, *MMSE* The Mini Mental State Examination

Another important aspect observed among older Portuguese immigrants is language barrier, as the Portuguese often is the only language, they are proficient in or comfortable to use. As a result, it can negatively influence their social relationships, feelings of belonging, and wellbeing [50]. Social participation in which individuals interact in their mother language can lead to an increase in social integration and has been shown to contribute to a healthier aging [50]. Therefore, the MEDITAGING offers the courses in Portuguese.

Regarding the scores on cognitive functioning, we exclude participants with Mini Mental State Examination (MMSE) below 22 because it is necessary to have a considerable level of insight to take advantage of the programs. Likewise, illiterate participants are excluded as reading and writing are indispensable to carry out the activities proposed in either one of the two groups.

## Recruitment

Coordinated communication strategies include spreading information about the MEDITAGING study to the Portuguese-speaking population, Portuguese Associations, local clinics, senior clubs, churches, brochures, and mass media (i.e., Facebook, newspaper advertising, radio programs, interviews). Communication materials are being made available in two languages (Portuguese and French). We have sent a printed letter to the institutions related to Portuguese-speaking people, informing them about the launch of the study and we are promoting lectures and booths at public events. With this mix of communication channels, we hope to reach our recruitment aim of 90 participants.

**Fig. 1:**
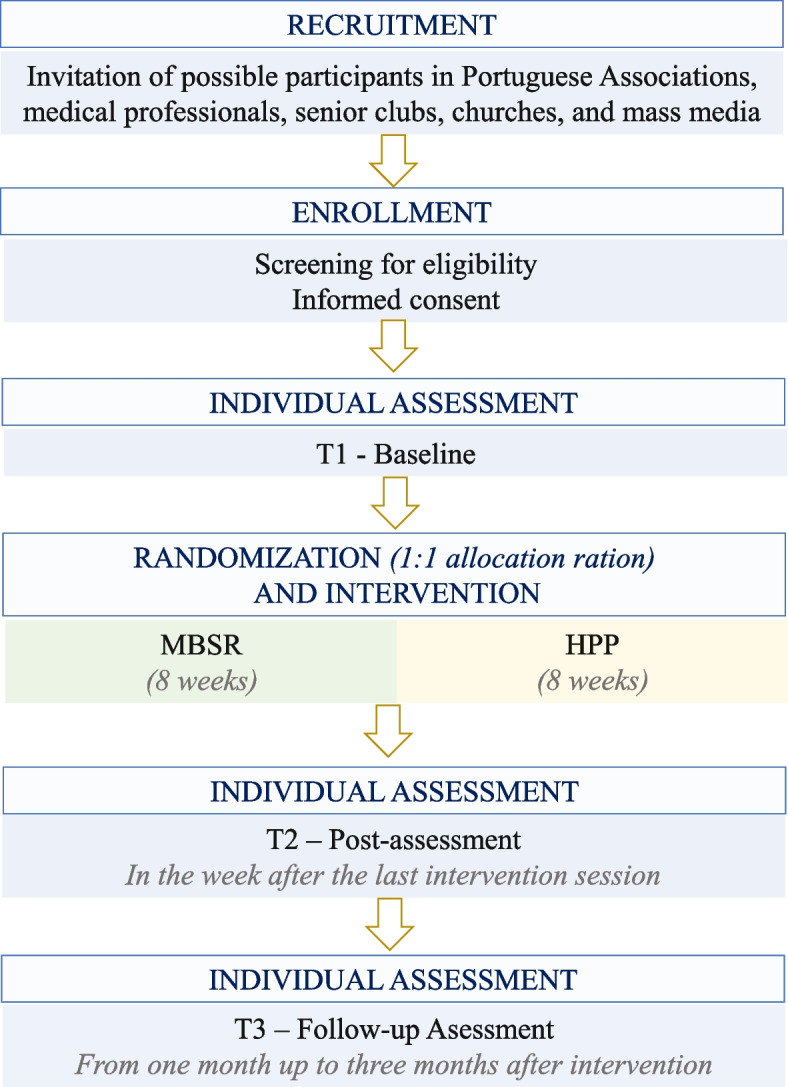
Study design and participant flow diagram. Note. MBSR Mindfulness based-stress-reduction, HPP Health promotion program

In case potential participants are interested in joining the program, ACT schedules an individual meeting, in which they are screened for eligibility and the informed consent is signed. During the first meeting, baseline assessments are performed, including sociodemographic and health-related questionnaires, cognitive tests, and qualitative interview). Participants also provide saliva for cortisol analysis and have their heart rate variability recorded. If any risk is identified (e.g., possibility of mild cognitive impairment), participants are informed about how to access specialist care services. Figure 1 and Table 2 provide a detailed description of the assessment timepoints. No financial compensation or inducements are provided to the subjects participating in this study.

**Table 2:** Screening and assessment measures

Measures	Screening	Baseline (T1)	Post intervention (T2)	Follow-up (T3)
Demographic and health questionnaire	x			
MMSE	x		x	x
DSM-5 Self-Rated Level 1 Cross-Cutting Symptom	x			
Saliva sample for cortisol analysis		x	x	
TMT		x	x	x
Stroop color word task		x	x	x
Letter-number sequencing		x	x	x
PSQI		x	x	x
PPS		x	x	x
MAAS		x	x	x
GAI		x	x	x
GDS-5		x	x	x
Heart rate variability		x	x	x
Semi-structured interviews		x	x	x

*DSM* Diagnostic and Statistical Manual of Mental Disorders, *GAI* Geriatric Anxiety Inventory, *GDS-5* Geriatric Depression 5 Item Scale, *MAAS* Mindfulness Attention and Awareness Scale, *MMSE* Mini Mental State Examination, *PPS* Perceived Stress Scale, *PSQI* Pittsburgh Sleep Quality Index, *TMT* Trail Making Test

### Informed consent

Informed consent is requested from the participants before they participate. A model of informed consent form will be obtained from the authors upon request. Participants confirm that they were provided with oral and written information about their participation, the nature, objectives, methods, as well as anticipated benefits and potential risks of the study. Additionally, by checking some boxes in the paper form, they authorize the research team to record, analyze, and use their data for scientific purposes and dissemination.

### Sample size estimation

No previous study has examined the MBSR cognitive effects on older migrant adults. However, a study with older adults reported a small MBSR effect size on attention [51]. Consequently, we assume a small effect (f = 0.14) and estimated the sample size using the G* Power – 3.1 statistical software [52] for repeated-measures ANOVA, considering within-between interaction (group x time interaction), with 80% of power and 5% of type I error probability. Taking an attrition rate of 5% reported in the mentioned study, we will need 90 participants, 45 in each arm.

### Intervention procedures

At the end of the baseline session, participants are randomized to one of two conditions: MBSR or health promotion program. After the intervention, there are two assessments: one immediately after the last program session and another during a follow-up session, ranging from one up to three months thereafter. This 3-month interval for the follow-up session is due to the fact that many participants frequently commute between Portugal and Luxembourg, which lead us to have a more spread interval for the assessments.

Both interventions, the MBSR and the health promotion program, were matched in terms of duration, home practice, a half day intensive practice of four hours, and a psychoeducational component with group discussion. The sessions are weekly delivered in face-to-face groups of around nine people, having the duration of two and a half hours each one. Participants are asked to practice for around 40 minutes at home, on a daily basis. Participants from both groups receive printed manuals regarding the contents and activities performed. The sessions of both groups are led by the first author ACT, who is a psychologist qualified to apply the MBSR program by the UC San Diego Center for Mindfulness. ACT has vast experience and training in group intervention, community therapy, teaching, and background in Psychology of Aging. FR led the watercolor session of the health promotion program. The general structure of both programs is presented in Table 3.

**Table 3:** Overview of the activities performed in both groups: MBSR and health promotion program

Session Number	Activities in each group	
	MBSR	Health promotion program
**1**	*Theme:* - Automatic pilot vs. mindfulness. *Practices:* - Participants’ presentation; - Agreements for the operation of the group; - Eating meditation with raisins; - Body scan; - Group discussion. *Home practice:* - Body scan (audio-guided); - The Nine Dot Puzzle; - Being mindful during a routine activity and a meal.	*Theme:* - Modifiable lifestyle factors of dementia. *Practices:* - Participants’ presentation; - Agreements for the operation of the group; - Warm-up dynamics with movement exercises and memory training; - Lecture on modifiable risk factors. *Home practice:* - The Nine Dot Puzzle; - Healthy Habits Agenda.
**2**	*Theme:* - The role of perception in shaping our reality. *Practices:* - Standing yoga; - Body Scan; - The Nine Dot Puzzle; - Discussion on perspective change; - Sitting meditation. *Home practice:* - Body scan (audio-guided); - Sitting meditation (audio-guided); - Pleasant events calendar; - Being mindful during a routine activity.	*Theme:* - Formation of healthy habits and self-care. *Practices:* - Review of the agreements for the operation of the group; - Warm-up dynamics with singing and movement exercises; - Group discussion on modifiable risk factors. *Home practice:* - Preparing a presentation for next class; - Sleep diary.
**3**	*Theme:* - There is more pleasure and power when we are present *Practices:* - Mindful Lying Down Yoga; - Mindful Walking; - Sitting meditation; - Pleasant events calendar. *Home practice:* - Alternating yoga and body scan; - Sitting meditation (audio-guided); - Unpleasant events calendar; - Identify an autopilot during the week.	*Theme:* - Healthy habits and Sleeping Hygiene. *Practices:* - Group discussion on healthy habits and perspective change; - Body movement; - Lecture on the Impact of Sleep on Mental and Physical Wellness. *Home practice:* - Having healthier habits; - Sleep diary.
**4**	*Theme:* - How conditioning and perception shape our experience. *Practices:* - Standing Yoga; - Sitting meditation; - Unpleasant events calendar; - Stressor naming; - Identification of body reaction to stress; - Awareness cycle. *Home practice:* - Body scan (audio-guided); - Lying down yoga (audio-guided); - Sitting meditation (audio-guided); - Be attentive to one stress reaction during the week.	*Theme:* - Sleep and Physical exercise; - Group discussion on sleep; - Physical exercises focusing on flexibility and strength. *Practices:* - Group discussion on healthy habits and on the Impact of Sleep on Mental and Physical Wellness. *Home practice:* - Having healthier habits; - Sleep diary.
**5**	*Theme:* - Responding skillfully to stress. *Practices:* - Standing yoga; - Sitting meditation; - Poem; - Reactivity and response cycle; - Strategies to coping with stress, considering a breathing space before responding. *Home practice:* - Sitting meditation, body scan and standing yoga (audio-guided); - Difficult communications calendar.	*Theme:* - Sleep hygiene and physical exercises. *Practices:* - Participant presentation about sleep hygiene tasks; - Physical exercises focusing on flexibility and strength. *Home practice:* - Physical exercises agenda.
**6**	*Theme:* - Stressful communications. *Practices:* - Standing yoga; - Sitting meditation; - Active listening practice; - Difficult communications calendar review; - Role Play. *Home practice:* - Alternate sitting meditation recording with body scan and/or standing or lying down Yoga; - World diet - how to take better care of oneself.	*Theme:* - Nutrition. *Practices:* - Body movements; - Lecture on eating well and label reading; - Discussion about nutrition, and food portions. *Home practice:* - Learning healthier recipes.
**Half-day class**	*Activity:* - Silence retreat. *Tasks:* - Yoga; - Body Scan; - Walking Meditation; - Mountain Meditation; - Eating Meditation; - Loving-Kindness Meditation; - Contemplation.	*Activity:* - Watercolor workshop. *Tasks:* - Learning watercolor techniques; - Learning the principle of primary, secondary, and tertiary colors; - Painting of one or two arts (an animal or a flower).
**7**	*Theme:* - Interacting with the World: Skillful Choices and Self-Care. *Practices:* - Yoga (with participants guiding); - Changing Seats: Exploring the familiar and the unfamiliar; - Sitting meditation, mostly silent; - Retreat discussion; - World diet – reviewing daily habits (invigorating and exhausting activities and exploring how to create a balance between them). *Home practice:* - Practice of sitting, yoga, walking and/or the body scan without recording; - Being as aware as possible throughout the day.	*Theme:* - Nutrition and revitalizing the mind. *Practices:* - Review of the nutritional habits; - Body movement exercises; - Attention training and discussion on how to stimulate cognition. *Home practice:* - Activities of cognitive stimulation.
**8**	*Theme:* - The Beginning of a Mindful Life. *Practices:* - Recognition of feeling; - Qi Cong massage; - Body scan; - Sitting meditation, mostly silent; - Poem; - Home practice discussion; - Letter to self and reflection on what participants have learned in the course; - Resources to sustain practice after the ending of the program. *Home practice:* - Accessing ongoing resources (eg., online, handouts, community, regional, internet); - Keeping up the practice creating a plan of practice for the participant daily routine.	*Theme:* - *Revitalize your mind and review the program.* *Practices:* - Cognitive training; - Positive behaviors; - Reflection on what participants have learned in the course.

*MBSR* mindfulness-based stress reduction program

#### Experimental condition – MBSR

The most used mindfulness-based training approach in Western societies is the MBSR [53, 54], which is the program we will apply in this study. It incorporates secular mindfulness meditation techniques into a structured program created to alleviate suffering through self-regulation mechanisms. We will follow the original program of Kabat-Zinn [11, 55], an eight-week group-based psychoeducational program with weekly meetings with a duration of 2.5 hours, a retreat, and at-home activities. Participants are trained to be mindful (i.e., to be attentive to the present moment and to their thoughts, feelings, and body sensations, being more able to identify automatic patterns and adopt more conscious and adaptive responses to the circumstances). Therefore, this training provides greater ability to monitor thoughts, noticing the wandering mind, and redirecting it to the object of interest, usually called anchor [56].

Participants practice acceptance and acknowledge whatever is present, including negative and unwanted experiences, feelings, and thoughts. They are also invited to be aware of the transitory and impersonality of the experiences of life. During the training, the importance of bringing mindfulness and the practices to life and to the daily routine is emphasized. Sessions usually start with formal mindfulness practices, which are followed by group discussion, review of home practices, and recommendation of new activities.

More specifically, the program techniques consist of guided body scan, hatha yoga, and different meditation techniques (e.g., sitting, eating, walking). Discussion about relevant topics related to the physiology of stress and strategies of coping with stress is also included as well as daily at-home practice six days a week, including daily informal practices (e.g., make one activity of daily life mindfully). For home practice, participants were provided with audios containing guidance of the recommended practice. Participants are asked to record their home practice indicating the activity performed and its duration. There is also a silent retreat towards the end of the program, during which meditation skills are addressed with different techniques. Participants have the opportunity for extended introspection during this period. The retreat of the MEDITAGING protocol have the duration of four hours since an all-day retreat could be too tiring for older participants. Modification in the protocol, especially in the retreat session to adapt to the necessity of participants, has also been executed in previous studies [57, 58], with a literature review suggesting that a reduction in the duration of the protocol does not reduce the effectiveness of the traditional intervention [57].

#### The health promotion program

The health promotion program is used as an active control condition, which is rarely applied in the studies in the field since most of them use a passive control condition (see [12, 16, 17]). The control group is important to monitor expectancy effects, such as the Hawthorne effect [59] and social interaction.

The health promotion program consists of a psychoeducation program focusing on the following topics: risk factors for dementia, healthy habits, sleep quality, nutrition, unhealthy behaviors, physical exercise, and cognitive stimulation. Multidomain interventions have been previously proved to be well accepted by participants and have an impact in lifestyle (e.g., physical activity, sleep quality, and alcohol use), as well as subjective well-being and cognition [60, 61]. Therefore, from an ethical point of view, participants from both programs benefit from participating in this study. Also, this kind of active control condition has been employed in previous mindfulness trials [62, 63].

The health promotion program follows the same structure of the MBSR program, but its sessions do not include mindfulness training. The discussions are followed by practical exercises and at-home activity. There is a four-hour watercolor workshop similar in length to the retreat held in the MBSR group. Handouts are provided with activities to be performed at home. We developed the activities of this group based on previous literature [9, 60, 62, 64, 65].

## Criteria for discontinuing intervention

All participants have the option to withdraw from the study and ask their samples and/or data to be destroyed at any time without the need to give a reason. However, whenever possible, reasons for dropping out will be recorded. In case adverse effects are identified, they will be explored and may result in the intervention being discontinued for the participants.

### Adherence and feasibility

A previous study has shown a dropout rate of 5% [51]. However, the dropout rate may be higher in the MEDITAGING project since 25.2% of the Portuguese immigrants plan to commute between Portugal and Luxembourg [66]. The adherence of home practice in a previous review has shown to be around 60% [67]. To increase adherence, weekly feedback will be requested from participants throughout the intervention, in order to detect and resolve any situation that needs attention.

Due to the under-researched target population of the MEDITAGING study, it has a secondary aim of investigating the operational feasibility of the MBSR for older migrants, as well as the financial, physical, and psychological feasibility of participating in the program. In particular, the following aspects will be evaluated: if participants both, those remaining in the study and those lost to follow-up, report [1] the need of any adjustment in the program [2]; any difficulties encountered (e.g., time restriction, physical limitation, anxiety, financial problems).

For the adherence and dropout rate analysis, the proportion of participants who completed the baseline assessment and went to the first group session and the number of participants that attended at least three sessions will be considered.

### Outcome measures

As outcome variables, we will assess executive functioning as primary outcome, as well as general cognitive ability, sleep quality, perceived stress, depression, anxiety, mindfulness-trait, cortisol level, and heart rate variability (HRV) as secondary ones. Additional exploratory analyses will be done to verify if the effects are moderated by demographic factors, such as gender and educational level, as well as number of attended sessions, stress, depression, mindfulness trait, anxiety, cortisol level, and sleep quality. We anticipate that the MBSR group will present gains (post-intervention compared to baseline) in cognitive tasks and stress relief and these gains will be higher than in the control group (health promotion program). A summary of the outcome measures is provided in Table 4. We aimed to select brief measures to minimize participant fatigue.

**Table 4:** Summary of primary and secondary outcomes

Outcome	Construct/surrogate	Description
***Primary outcomes***		
TMT (Cavaco et al., 2013)	Executive functions	TMT Part A evaluates attention, visual scanning, the speed of eye-hand coordination, and information processing. Part B assesses working memory and executive functions associated with the capacity to switch between sets of stimuli. The resulting scores include difference (B-A), ratio (B/A), and proportion (B-A/A).
Stroop color word task (Golden, 1978)	Selective attention, inhibitory control, and cognitive flexibility	The Stroop Test evaluates cognitive flexibility and attention span through a series of three distinct subtests. In each subtest, there are 100 items organized in 5 columns with 20 items per column. The time allocated for each subtest is limited to 45 seconds. In the initial subtest, individuals are instructed to verbally read aloud the color names (such as red, green, or blue) that are written in black ink. For the second subtest, participants are presented with three letters “X” printed in red, green, or blue and are required to state the color of the ink. In the third subtest, subjects are asked to announce the color of the ink in which the words are printed (for instance, if the word “blue” is printed in green ink, the individual should say “green”). The time taken to complete each task and the number of errors made are recorded. The total score is calculated as the sum of scores from all three subtests, or alternatively, an interference index can be determined by subtracting the time taken to complete the color-naming trial from the word-reading trial.
Letter-number sequencing from WAIS-III (Wechsler, 2008)	Working memory	In the Letter-Number Sequencing task, individuals are presented with a series containing a mix of letters and numbers. Their task involves repeating these sequences by initially stating the numbers in ascending order and subsequently reciting the letters in alphabetical order. The score is determined by the count of correctly manipulated lists.
***Secondary outcomes***		
PSQI (del Rio João et al., 2017)	Sleep quality	The PSQI is a questionnaire comprising 19 items designed to assess sleep quality and disruptions over a one-month period. There are five supplementary questions that require responses from the participant’s roommate. These questions are divided into seven distinct categories: 1) subjective sleep quality, 2) sleep latency, 3) sleep duration, 4) usual sleep efficiency, 5) sleep disturbances, 6) use of sleeping medication, and 7) daytime dysfunction. The cumulative scores from these categories yield a global score ranging from 0 to 21, with a higher score indicating poorer sleep quality. An overall PSQI score exceeding 5 suggests significant difficulties in at least two of these categories or moderate difficulties in more than three of them.
PPS (Mota-Cardoso et al., 2002)	Perceived stress	The PSS is a five-point Likert scale consisting, designed to evaluate the individual’s perception of stress. In its Portuguese version, it comprises 10 items. Participants are asked to assess the frequency of their emotions and thoughts concerning situations that have arisen in the past month. Responses range from “never” to “very often.” Four of the items are positively worded and are subsequently reverse scored before being combined with the negatively worded items. The resulting score falls within a range of 0 to 40, with a higher score indicating a heightened level of stress perception.
MAAS (Gregório & Pinto-Gouveia, 2013)	Dispositional quality of mindfulness	The MAAS evaluates the dispositional aspect of mindfulness, particularly focusing on the attention and awareness that individuals have in their daily life experiences. This assessment comprises 15 items, and the overall score is derived by summing the individual scores. The scoring scale ranges from 15 to 90, with higher scores indicating a higher degree of mindfulness.
GAI (Ribeiro et al., 2011)	Anxiety symptoms	The GAI is a tool designed to evaluate anxiety symptoms in older individuals. It comprises a set of 20 statements where respondents indicate their agreement or disagreement. The recommended threshold for identifying severe anxiety symptoms is when the score reaches 8 or 9.
GDS-5 (Santos et al., 2019)	Depressive symptomatology	The GDS-5 represents a condensed version of the Geriatric Depression Scale, consisting of only five items. It serves as a screening tool for detecting depressive symptoms among older individuals. In the Portuguese adaptation, there are only four items and they are scored with either 0 or 1. The overall score is derived by summing these items, and the suggested cutoff point for identifying depressive symptoms is set at 2 points.
MMSE (Freitas et al., 2015)	General cognitive state	The MMSE is a brief screening assessment that offers a concise evaluation of cognitive abilities. It encompasses various tasks to assess orientation regarding time and place, the ability to register information, attention and calculation skills, spontaneous recall, language proficiency (including naming, repetition, reading, and spontaneous writing), and visual construction aptitude. Participants receive one point for each correct response, with a maximum achievable score of 30 points upon completion.
Salivary Cortisol	Stress marker	Saliva samples are gathered for cortisol analysis through the utilization of Salivette assays. These samples are obtained immediately upon waking, as well as at 30 minutes and eight hours after awakening. Various indices related to diurnal cortisol measurements, such as the peak cortisol level and the area under the curve concerning the baseline, will be thoroughly examined (Fekedulegn et al., 2007; Lenze et al., 2012).
Resting heart rate variability - HRV (Kim et al., 2018)	Surrogate for psychological health and stress	HRV pertains to the fluctuations in the duration of intervals between heartbeats. It is closely linked to the heart’s capacity to respond to a range of physiological and environmental stimuli. Reduced HRV is associated with compromised regulation and homeostatic functions of the autonomic nervous system, which in turn diminishes the body’s ability to handle stressors effectively. One of the simplest time-domain analysis metrics is the standard deviation of the NN interval (SDNN). A higher SDNN value indicates a greater level of HRV. Another commonly utilized index is the root mean square of the successive differences (RMSSD), which is essentially the square root of the mean of the sum of the squares of differences between adjacent NN intervals.
***Screening***		
Sociodemographic and health-related information	Sociodemographic and health-related information	It is a structured interview in which participants answered questions regarding age, gender, years of formal education, country of origin, number of years living in Luxembourg, mother language, marital status, professional occupation, people living in the household, disease history, surgical interventions, pharmacological treatments, health complaints, difficulties in daily life, psychological intervention, mindfulness or meditative practices, religion, physical exercise, weight, and height.
DSM-5 Self-Rated Level 1 Cross-Cutting Symptom (Bastiaens & Galus, 2018)	Assessment of psychopathology	The DSM-5 is a screening tool to assess various forms of psychopathology, including depression, anger, mania, anxiety, somatic symptoms, suicidal ideation, psychosis, sleep disturbances, memory issues, repetitive thoughts and behaviors, dissociation, personality functioning, and substance use. For this specific protocol, we have chosen to focus solely on items related to the domains of mania, suicidal ideation, psychosis, and substance use. Each item is evaluated using a 5-point scale, where 0 represents the absence of symptoms or no occurrence, 1 indicates mild or infrequent symptoms occurring for less than 2 days, 2 denotes mild symptoms experienced over several days, 3 signifies moderate symptoms occurring on more than half of the days, and 4 indicates severe symptoms experienced nearly every day. A rating of 2 or higher is considered a potential indicator for further investigation. However, for substance use and suicidal ideation, a rating of 1 is used as the cutoff point for considering additional inquiry.
**Qualitative outcome**		
Semi-structured interview	Subjective effects and feasibility	Semi-structured interview asking about motivation, expectations, the need of any adjustment in the program, the difficulties that participants struggled during the program (e.g., time restriction, physical limitation, anxiety, financial problems), if participants realized any change in their daily life due to the program and so were motivated to keep practicing during and after the program, if the group was supportive for the development of the program, if participants keep using the techniques learned at the follow-up session, perceived effects, and suggestions.

*GAI* Geriatric Anxiety Inventory, *GDS-5* Geriatric Depression 5 Item Scale, *HRV* Heart rate variability, *MAAS* The Mindfulness Attention and Awareness Scale, *MMSE* The Mini Mental State Examination, *PPS* Perceived Stress Scale, *PSQI* Pittsburgh sleep quality index, *TMT* Trail making test

Previous reviews have shown that mindfulness may improve executive attention (mental set shifting and inhibition), as well as working memory, but overall evidence remains limited [32, 56, 68]. One study has shown the recruitment of attentional control regions such as the prefrontal cortex and anterior cingulate cortex in participants during meditation activities [69]. Additionally, older adults have shown to benefit of mindfulness in cognition, especially on attention regulation [70, 71]. Therefore, primary outcomes will be the executive functioning measures, namely the score on TMT, Stroop color word task, and letter-number sequencing from WAIS-III [72].

We will run exploratory secondary analyses including the following measures: the Pittsburgh sleep quality index (PSQI), Perceived Stress Scale (PPS), Mindfulness Attention and Awareness Scale (MAAS), Geriatric Anxiety Inventory (GAI), Geriatric Depression 5 Item Scale (GDS-5), the Mini Mental State Examination (MMSE) scores, as well as cortisol levels and heart rate variability (HRV). Cortisol level was only assessed at baseline and during post-intervention assessment (not in follow-up session, as the financial resources are limited). For the self-reported measures, the total score for the scale and subscales will be used as outcomes.

A cortisol test measuring the diurnal cortisol cycle is done with saliva samples collected at the participants’ home following manufacturer’s instructions (Salivette®, Sarstedt, Nümbrecht, Germany). Saliva collection is self-administered before and upon completion of the intervention, at three time points: 1) upon waking up, 2) 30 minutes after waking up, and 3) at 8 hours after waking up. Sarstedt salivette® kits are delivered to participants at baseline and after the intervention. Participants receive explanations on how to collect the saliva, specifically, participants are told to remove the swab from the Salivette® and place it in the mouth for about 120 seconds. Then, they return the swab to the Salivette®, replace the stopper, and store the sample in the home fridge. Normally, up to 1 day after saliva collection, participants themselves bring the collected samples to the place of intervention and give them to ACT who brings the samples to the laboratory for processing, analysis, and storage. At the laboratory, salivettes are stored frozen for up to 7 days (the tests are done once a week). Samples are centrifuged at 1000 rpm and aliquots are analysed by ELISA (Demeditec Saliva Free Cortisol Kit – Demeditec DES6611, Demeditec Diagnostics GmbH, Kiel, Germany) following the user manual. The samples are diluted if the results are greater than 30 ng/ml.

Heart rate (HR) assessments are performed individually during rest at baseline, post-test, and follow-up sessions. Participants comfortably sit using a Polar H10 chest strap and are instructed to lean back in the chair, to relax, and avoid movement for 15 minutes. In the last 5 minutes, HR is recorded using the Elite HRV smartphone application (Elite HRV Inc., Asheville, NC, USA). Each HR measure is recorded at two different times on the same day.

### Randomization and blindness

At the end of the baseline assessment, participants are randomized to the MBSR or the health promotion program intervention. The randomization list was generated through a website (http://www.randomization.com) in blocks of 10 participants with a ratio of 1:1 to receive the MBSR or the health promotion program (i.e., five individuals randomly allocated to each group). The allocation list is masked from all study personnel. The condition of each participant is described in different excel sheets in a way that the researcher responsible for the randomization would only have access to the allocation of the next participant (allocation concealment). Assessors and participants are blinded to the conditions prior to randomization. During the intervention, although participants know which activity they are included in, they do not know which the experimental or control conditions are. If participants want to know about our hypotheses, they may be disclosed after the study is completed. To test participants’ blinding, they fill out a questionnaire of credibility and expectancy adapted from Devilly and Borkovec [73]. Due to the researchers’ availability, the intervention facilitator and the data analyst are not blinded for participants’ conditions since it is performed by the first author of this study (ACT).

### Management of data collection

Training is provided for all assessors in order to guarantee the quality of the assessment and the safety of participants. Adverse effects of the interventions are assessed through the Positive and Negative Affect Schedule (PANAS) and a Visual Analog Scale (VAS), administered in five different sessions of the project, as well as by the interview performed during the post-intervention session. This allows us to identify any unwanted effects associated with the MBSR. Although the risks related to the participation in this study are minimal, ACT, JS, and AKL periodically review the adverse effects and make decisions when they are verified. Changes to the study protocol will be updated accordingly in clinicaltrials.gov. ACT has the responsibility to conduct and monitor the necessary tasks to run the study. A monthly meeting is held to discuss the quality and development of the project, intercalated with specific meetings focusing on specific tasks and aims of the project.

Most of the information is collected on paper and will be stored in a database. All samples and data are pseudonymized. Only one researcher (ACT) and the neuropsychologist assessor are able to match the participants’ name with their data. Names are replaced by codes from which identification is impossible. No personally identifiable information is transferred to the laboratory that process and analyze the saliva samples. All data collected will be entered into an eCRF system (Redcap) hosted under control of the researchers (ACT and AKL) acting as the Data Controller. The database of the eCRF system is protected by the firewall and has secure online access for the investigators. Data quality is checked by a second person not responsible for data entry. No Data Monitoring Committee is designated to this study, which is justified given it is a low-risk intervention.

### Statistical analyses

Regarding statistical analyses, data will be explored for missing and impossible values and descriptive statistics will be provided. The analyzes will be based on the modified intention-to-treat (mITT) population (i.e., participants that participated in at least three group-sessions and have at least one post-intervention assessment). We chose three sessions based on previous studies showing cognitive changes after three sessions of cognitive training [74]. A level of significance of less than 5% will be used in the analyses.

We will use mixed-effect regression models with maximum likelihood estimation to analyze change in cognitive scores as a function of the interaction group*time and participants will be treated as a random effect. *P*-value will be set at *p* < .05. Effect sizes (Hedges, *g*) will be calculated for each score [75–77]. Demographic factors, such as gender and educational level, the number of attended sessions, as well as the score differences in stress relief, depression, anxiety, mindfulness state, and sleep quality will be analyzed by running separate models for each factor.

Concerning the interview, it will be recorded in audio and transcribed in Portuguese by a member of the research team. The interview data will be analyzed according to Malterud’s principles of systematic text condensation and guidelines for qualitative research [78]. Content analysis can be conducted using the software package NVivo 12 [79].

### Reporting and dissemination

Results of the MEDITAGING project will be published in scientific journals and conferences. All scientific contributors will be included as authors according to international authorship guidelines. Only aggregated data will be published. Furthermore, specific scientific talks open to the public are being organized, with the entities participating in the study, to present the main findings to the participants, as well as to give them strategies to promote the successful aging. We will also take advantage of the mass media (e.g., newspapers, Facebook, Twitter, websites) to share findings with the scientific community and the public.

## Discussion

The study protocol was developed to provide good quality interventions to promote active aging among vulnerable older immigrants, especially focusing on cognition, stress reduction, and social interaction. Luxembourg offers a suitable context to run the MEDITAGING study since many immigrants, especially Portuguese speakers, are nowadays older adults with less favorable social participation profiles [8]. Consequently, older migrants may be more vulnerable to physical and mental disorders, which may come with increased needs for health and social care.

The MEDITAGING study aims to compare the effects of two group-programs (MBSR and health promotion program) offered to Portuguese-speaking immigrants aged 55 or over, living in Luxembourg and frontier regions. Other secondary aims such as characterization of the cognitive functioning and emotional state of the participants are also planned. Regarding the interventions, the MBSR follows the traditional curriculum [55], while the health promotion program was made following the literature in the field [9, 60, 62, 64]. As we developed an innovative control condition, we address the ethical and methodological challenge of providing health promotion education to the control group as well, in contrast to earlier studies that used a passive control condition only (see [12, 16, 17, 65]). Due to the active control condition [15–18], we believe that both intervention groups will benefit from the interventions. Nonetheless, due to the nature of the intervention condition, we hypothesize that the MBSR group will present more gains in executive functioning in line with the intention of the program [11]. Additionally, we expect that the MBSR group will present gains in stress relief, which will also be reflected in lower cortisol levels [80] and higher heart rate variability [81]. These surrogate measures are important to complement the neuropsychological assessment and questionnaires, supporting evidence for the efficacy of the interventions [82].

Furthermore, there is a paucity of evidence regarding mindfulness training effects in older adults, especially with vulnerable populations (see [83] for a recent review). This lack of studies was demonstrated in a review suggesting that, in high-income countries, there are few programs promoting health in disadvantaged populations [84].

Regarding the limitations, interventions with other migrant groups may be needed to understand if the results of MEDITAGING can be generalized. Second, written assignments that are part of the MBSR may be less effective in the addressed population, as most of the participants have low levels of education and do not feel comfortable writing. It would be important that future studies use other modalities of registration, such as recording, to register their answers and practices. Third, Some of the participants are excluded due to illiteracy, as the MBSR and health promotion materials require reading skills. Inclusion of illiterate people in future studies is important to test effects in this even more vulnerable group. Fourth, the study may lack power since we have based our sample size calculation in a study with higher educated population and effects may be smaller in our target population [26]. No other study with a similar population and endpoints was published by the time we planned this project. Fifth, the high level of sickness days due to the pandemic and the frequent travels to the origin countries of the participants may lead to a high dropout rate. Sixth, differences in outcomes between intervention and control group may be rather small, since participants in the control condition are receiving an intervention to promote a healthier lifestyle, which is supposed to improve lifestyle, well-being, cognition, and protect against dementia [60, 61, 85]. Finally, it would also be important to follow-up participants for a longer period of time to evaluate if the effects are maintained in the long-term.

## Conclusion

We believe that MEDITAGING is an important initiative contributing to the evidence base on non-pharmacological interventions aimed at promoting healthy aging, especially among vulnerable populations such as migrants. Feedback from national partners suggests that initiatives like the MEDITAGING project fill a need related to immigration. The diversity and singularity of older migrants needs to be addressed in interventions like this, as well as incorporated in public health policies [7, 8, 86].

### Supplementary Information

**Additional file 1.**

## Data Availability

Data supporting our conclusions may be available from the corresponding author on reasonable request.

## Abbreviations

MBSR: mindfulness based-stress reduction
HPP: Health promotion program

## References

[1] Burch JB, Augustine AD, Frieden LA, Hadley E, Howcroft TK, Johnson R, et al. Advances in geroscience: Impact on healthspan and chronic disease. J Gerontol-Series A Biol Sci Med Sci. 2014;69 10.1093/gerona/glu041 PMC4036419 24833579

[2] Chaves AS, Santos AM dos, Alves MTSS de B e, Salgado Filho N. Associação entre declínio cognitivo e qualidade de vida de idosos hipertensos. Revista Brasileira de Geriatria e Gerontologia. 2015 18(3):545–56 10.1590/1809-9823.2015.14043

[3] Pusswald G, Tropper E, Kryspin-Exner I, Moser D, Klug S, Auff E, et al. Health-related quality of life in patients with subjective cognitive decline and mild cognitive impairment and its relation to activities of daily living. J Alzheimers Dis. 2015; 10.3233/JAD-150284 26401569

[4] Selten JP, Termorshuizen F, van Sonsbeek M, Bogers J, Schmand B (2021). Migration and dementia: a meta-analysis of epidemiological studies in Europe. Psychol Med..

[5] Maleku A, España M, Jarrott S, Karandikar S, Parekh R (2022). We are aging too! Exploring the social impact of late-life migration among older immigrants in the United States. J Immigr Refug Stud..

[6] Statec (2020). Luxembourg in figures.

[7] Zahlen P (2018). Elderly migrants in Luxembourg. Ageing in Contexts of Migration.

[8] Ramos AC, Karl U (2016). Social relations, Long-Term Care and Well-Being of Older Migrants in Luxembourg.

[9] Livingston G, Huntley J, Sommerlad A, Ames D, Ballard C, Banerjee S, et al. Dementia prevention, intervention, and care: 2020 report of the lancet commission. Lancet. 2020; 10.1016/S0140-6736(20)30367-6 PMC7392084 32738937

[10] Livingston G, Sommerlad A, Orgeta V, Costafreda SG, Huntley J, Ames D, et al. Dementia prevention, intervention, and care. Lancet. 2017; 10.1016/S0140-6736(17)31363-6 28735855

[11] Kabat-Zinn J. Full catastrophe living:Using the wisdom of your body and mind to face stress, pain, and illness. The Nurse Pract. 1992;

[12] Baer RA. Mindfulness training as a clinical intervention: A conceptual and empirical review 10. Clin Psychol: Sci Pract. 2003;

[13] Bishop SR, Lau M, Shapiro S, Carlson L, Anderson ND, Carmody J (2004). Mindfulness: A proposed operational definition. Clin Psychol Sci Pract..

[14] Moore A, Malinowski P. Meditation, mindfulness and cognitive flexibility. Conscious Cogn. 2009;18(1) 10.1016/j.concog.2008.12.008 19181542

[15] Fountain-Zaragoza S, Prakash RS. Mindfulness training for healthy aging: impact on attention, well-being, and inflammation. Front aging Neurosci. 2017; 10.3389/fnagi.2017.00011 PMC5289973 28217093

[16] Gard T, Hölzel BK, Lazar SW. The potential effects of meditation on age-related cognitive decline: A systematic review. Ann N Y Acad Sci. 2014; 10.1111/nyas.12348 PMC4024457 24571182

[17] Gill LN, Renault R, Campbell E, Rainville P, Khoury B. Mindfulness induction and cognition: A systematic review and meta-analysis. Conscious Cogn. 2020; 10.1016/j.concog.2020.102991 32739799

[18] Pandya SP. Meditation to improve the quality of life of community-dwelling ever-single older adults: A multi-city five-year follow-up experiment. J Relig Spiritual Aging. 2020;

[19] Whitfield T, Barnhofer T, Acabchuk R, Cohen A, Lee M, Schlosser M, et al. The effect of mindfulness-based programs on cognitive function in adults: A systematic review and Meta-analysis. Neuropsychol Rev. 2021; 10.1007/s11065-021-09519-y PMC9381612 34350544

[20] Mirabito G, Verhaeghen P (2023). The effects of mindfulness interventions on older adults’ cognition: A Meta-analysis. J Gerontol: Series B..

[21] Cornelis MC, Wang Y, Holland T, Agarwal P, Weintraub S, Morris MC. Age and cognitive decline in the UK biobank. PLoS One. 2019;14(3) 10.1371/journal.pone.0213948 PMC6422276 30883587

[22] Jha AP, Denkova E, Zanesco AP, Witkin JE, Rooks J, Rogers SL. Does mindfulness training help working memory ‘work’ better? Curr Opin Psychol. 2019;28 10.1016/j.copsyc.2019.02.012 30999122

[23] Teixeira-Santos AC, Moreira CS, Magalhães R, Magalhães C, Pereira DR, Leite J, et al. Reviewing working memory training gains in healthy older adults: A meta-analytic review of transfer for cognitive outcomes. 2019;103:163–77. Available from: http://www.ncbi.nlm.nih.gov/pubmed/31100297 10.1016/j.neubiorev.2019.05.009 31100297

[24] Fox KCR, Nijeboer S, Dixon ML, Floman JL, Ellamil M, Rumak SP, et al. Is meditation associated with altered brain structure? A systematic review and meta-analysis of morphometric neuroimaging in meditation practitioners. Neurosci Biobehav Rev. 2014; 10.1016/j.neubiorev.2014.03.016 24705269

[25] Hölzel BK, Carmody J, Vangel M, Congleton C, Yerramsetti SM, Gard T, et al. Mindfulness practice leads to increases in regional brain gray matter density. Psychiatry Res Neuroimag. 2011; 10.1016/j.pscychresns.2010.08.006 PMC3004979 21071182

[26] Moynihan JA, Chapman BP, Klorman R, Krasner MS, Duberstein PR, Brown KW, et al. Mindfulness-based stress reduction for older adults: effects on executive function, frontal alpha asymmetry and immune function. Neuropsychobiol. 2013; 10.1159/000350949 PMC3831656 23774986

[27] O’Leary K, O’Neill S, Dockray S. A systematic review of the effects of mindfulness interventions on cortisol 21, Journal of Health Psychology. SAGE Publications Ltd; 2016 2108–2121. Available from: https://pubmed.ncbi.nlm.nih.gov/25673371/10.1177/1359105315569095 25673371

[28] Sharma M, Rush SE (2014). Mindfulness-based stress reduction as a stress management intervention for healthy individuals. J Evid Based Complementary Altern Med..

[29] Khoury B, Sharma M, Rush SE, Fournier C (2015). Mindfulness-based stress reduction for healthy individuals: A meta-analysis. J Psychosom Res..

[30] Cásedas L, Pirruccio V, Vadillo MA, Lupiáñez J (2020). Does mindfulness meditation training enhance executive control? A systematic review and Meta-analysis of randomized controlled trials in adults. Mindfulness (N Y).

[31] Im S, Stavas J, Lee J, Mir Z, Hazlett-Stevens H, Caplovitz G (2021). Does mindfulness-based intervention improve cognitive function?: A meta-analysis of controlled studies. Clin Psychol Rev..

[32] Lao SA, Kissane D, Meadows G (2016). Cognitive effects of MBSR/MBCT: A systematic review of neuropsychological outcomes. Conscious Cogn..

[33] Winbush NY, Gross CR, Kreitzer MJ (2007). The effects of mindfulness-based stress reduction on sleep disturbance: A systematic review. Explore..

[34] Lenze EJ, Voegtle M, Miller JP, Ances BM, Balota DA, Barch D (2022). Effects of mindfulness training and exercise on cognitive function in older adults. JAMA..

[35] Palta P, Page G, Piferi RL, Gill JM, Hayat MJ, Connolly AB, et al. Evaluation of a mindfulness-based intervention program to decrease blood pressure in low-income African-American older adults. J Urban Health. 2012; 10.1007/s11524-011-9654-6 PMC3324609 22302233

[36] Salthouse TA (2018). Trajectories of Normal cognitive aging. Psychol Aging..

[37] O’Hara R, Beaudreau SA, Luzon A, Hah M, Hubbard JT, Sommer B (2010). Stress, resilience, and the aging brain. Successful cognitive and emotional aging.

[38] Ouanes S, Popp J. High cortisol and the risk of dementia and alzheimer’s disease: A review of the literature. Front Aging Neurosci. 2019;11 10.3389/fnagi.2019.00043 PMC6405479 30881301

[39] Graham JE, Christian LM, Kiecolt-Glaser JK. Stress, age, and immune function: toward a lifespan approach. J Behav Med. 2006;29 10.1007/s10865-006-9057-4 PMC2805089 16715331

[40] McEwen BS. Central effects of stress hormones in health and disease: understanding the protective and damaging effects of stress and stress mediators. Eur J Pharmacol. 2008;583 10.1016/j.ejphar.2007.11.071 PMC2474765 18282566

[41] McEwen BS, Gianaros PJ (2011). Stress- and Allostasis-induced brain plasticity. Annu Rev Med..

[42] Prenderville JA, Kennedy PJ, Dinan TG, Cryan JF. Adding fuel to the fire: the impact of stress on the ageing brain. Trends Neurosci. 2015;38 10.1016/j.tins.2014.11.001 25705750

[43] Felsted KF. Mindfulness, stress, and aging. Clin Geriatr Med. 2020;36 10.1016/j.cger.2020.06.010 33010903

[44] Rojiani R, Santoyo JF, Rahrig H, Roth HD, Britton WB. Women benefit more than men in response to college-based meditation training. Front Psychol. 2017; 10.3389/fpsyg.2017.00551 PMC5397480 28473783

[45] Wong WP, Hassed C, Chambers R, Coles J. The Effects of Mindfulness on Persons with Mild Cognitive Impairment: Protocol for a Mixed-Methods Longitudinal Study. 2016;8(June):1–15. 10.3389/fnagi.2016.00156 PMC4923201 27445799

[46] Teixeira-Santos ACAC, Pinal D, Pereira DRDR, Leite J, Carvalho S, Sampaio A (2020). Probing the relationship between late endogenous ERP components with fluid intelligence in healthy older adults. Sci Rep..

[47] Teixeira-Santos AC, Moreira CS, Pereira DR, Pinal D, Fregni F, Leite J (2022). Working memory training coupled with transcranial direct current stimulation in older adults: A randomized controlled experiment. Front Aging Neurosci..

[48] Vøllestad J, Sivertsen B, Nielsen GH. Mindfulness-based stress reduction for patients with anxiety disorders: evaluation in a randomized controlled trial. Behav Res Ther. 2011;49(4) 10.1016/j.brat.2011.01.007 21320700

[49] Singh-Manoux A, Kivimaki M, Glymour MM, Elbaz A, Berr C, Ebmeier KP, et al. Timing of onset of cognitive decline: Results from Whitehall II prospective cohort study. BMJ. 2012;344(7840) 10.1136/bmj.d7622 PMC3281313 22223828

[50] Pot A, Keijzer M, de Bot K (2020). The language barrier in migrant aging. Int J Biling Educ Biling..

[51] Gallegos AM, Hoerger M, Talbot NL, Krasner MS, Knight JM, Moynihan JA, et al. Toward identifying the effects of the specific components of mindfulness-based stress reduction on biologic and emotional outcomes among older adults. J Altern Complement Med. 2013;19(10) 10.1089/acm.2012.0028 PMC3804316 23383976

[52] Faul F, Erdfelder E, Lang AG, Buchner A (2007). G*power 3: A flexible statistical power analysis program for the social, behavioral, and biomedical sciences. Behav Res Methods..

[53] Kabat-Zinn J (1982). An outpatient program in behavioral medicine for chronic pain patients based on the practice of mindfulness meditation: theoretical considerations and preliminary results. Gen Hosp Psychiatry..

[54] Kabat-Zinn J, Lipworth L, Burney R, Sellers W. Four-year follow-up of a meditation-based program for the self-regulation of chronic pain: treatment outcomes and compliance. Clin J Pain. 1986;

[55] Santorelli S, Meleo-Meyer F, Koerbel L, Kabat-Zinn J (2017). Mindfulness-based stress reduction (MBSR): authorized curriculum guide. Center for Mindfulness in Medicine, Health Care & Society.

[56] Gallant SN. Mindfulness meditation practice and executive functioning: breaking down the benefit. Conscious Cogn. 2016;40 10.1016/j.concog.2016.01.005 26784917

[57] Carmody J, Baer RA. How long does a mindfulness-based stress reduction program need to be? A review of class contact hours and effect sizes for psychological distress. J Clin Psychol. 2009;65 10.1002/jclp.20555 19309694

[58] Pereira DR, Silva ER, Carvalho-Maia C, Monteiro-Reis S, Lourenço C, Calisto R, et al. The modulatory role of internet-supported mindfulness-based cognitive therapy on extracellular vesicles and psychological distress in people who have had cancer: a protocol for a two-armed randomized controlled study. Trials. 2022;23(1) 10.1186/s13063-022-06045-x PMC8817152 35123569

[59] Wickstrom G, Bendix T. The “Hawthorne effect” - what did the original Hawthorne studies actually show? Scand J Work Environ Health. 2000;26:363–7. 10994804

[60] Mace RA, Greenberg J, Stauder M, Reynolds G, Vranceanu AM. My healthy brain: a multimodal lifestyle program to promote brain health. Aging Ment Health. 2022;26(5) 10.1080/13607863.2021.1904828 33784902

[61] Ngandu T, Lehtisalo J, Solomon A, Levälahti E, Ahtiluoto S, Antikainen R, et al. A 2 year multidomain intervention of diet, exercise, cognitive training, and vascular risk monitoring versus control to prevent cognitive decline in at-risk elderly people (FINGER): A randomised controlled trial. Lancet. 2015;385(9984) 10.1016/S0140-6736(15)60461-5 25771249

[62] MacCoon DG, Imel ZE, Rosenkranz MA, Sheftel JG, Weng HY, Sullivan JC (2012). The validation of an active control intervention for mindfulness based stress reduction (MBSR). Behav Res Ther.

[63] Marchant NL, Barnhofer T, Klimecki OM, Poisnel G, Lutz A, Arenaza-Urquijo E (2018). The SCD-well randomized controlled trial: effects of a mindfulness-based intervention versus health education on mental health in patients with subjective cognitive decline (SCD). Alzheimer’s and Dementia: Translational Research and Clinical Interventions.

[64] Lorig Kate D, Laurent Diana MPH, Gonzalez Virgina MPH, Sobel MPH, David MD, Minor PD, Marion PT, Gecht-Silver OTD, Maureen MPH (2020). Living a healthy life with chronic conditions: self-management skills for heart disease Arthritis Diabetes, Depression, Asthma, Bronchitis, Emphysema and Other Physical and Mental Health Conditions. Living a Healthy Life with Chronic Conditions.

[65] Johnson D (2023). A good night’s sleep: three strategies to rest, relax and restore energy. J Interprof Educ Pract..

[66] Albert I, Coimbra SB, Ferring D. Social inquiry into well-being future plans and the regulation of well-being of older Portuguese immigrants in Luxembourg. Soc Inq Well Being. 2016;

[67] Parsons CE, Crane C, Parsons LJ, Fjorback LO, Kuyken W. Home practice in mindfulness-based cognitive therapy and mindfulness-based stress reduction: A systematic review and meta-analysis of participants’ mindfulness practice and its association with outcomes. Behav Res Ther. 2017:95. 10.1016/j.brat.2017.05.004 PMC5501725 28527330

[68] Chiesa A, Serretti A. Mindfulness-based interventions for chronic pain: A systematic review of the evidence. J Altern Complement Med. 2011;17 10.1089/acm.2009.0546 21265650

[69] Tang YY, Hölzel BK, Posner MI. The neuroscience of mindfulness meditation. Nat Rev Neurosci. 2015; 10.1038/nrn3916 25783612

[70] Chételat G, Lutz A, Klimecki O, Frison E, Asselineau J, Schlosser M (2022). Effect of an 18-month meditation training on regional brain volume and perfusion in older adults. JAMA Neurol..

[71] Oken BS, Fonareva I, Haas M, Wahbeh H, Lane JB, Zajdel D, et al. Pilot controlled trial of mindfulness meditation and education for dementia caregivers. J Altern Complement Med. 2010;16(10) 10.1089/acm.2009.0733 PMC3110802 20929380

[72] Wechsler D. WAIS-III: Escala de inteligência de Wechsler para adultos-Terceira edição. Manual técnico. [WAIS-III: Wechsler adult intelligence scale - 3rd edition. Technical manual]. Lisboa: Cegog-Tea; 2008.

[73] Devilly GJ, Borkovec TD (2000). Psychometric properties of the credibility/expectancy questionnaire. J Behav Ther Exp Psychiatry.

[74] Borella E, Carretti B, Riboldi F, de Beni R (2010). Working memory training in older adults: evidence of transfer and maintenance effects. Psychol Aging..

[75] Cohen J (1988). Statistical Power Analysis for the Behavioural Science. Statistical Power Anaylsis for the Behavioral Sciences.

[76] Hedges L, v. (1989). An unbiased correction for sampling error in validity generalization studies. J Appl Psychol..

[77] Morris SB (2008). Estimating effect sizes from pretest-posttest-control group designs. Organ Res Methods..

[78] Malterud K (2012). Systematic text condensation: A strategy for qualitative analysis. Scand J Public Health..

[79] QSR International Pty Ltd. NVivo Qualitative Data Analysis Software 12 Pro. https://www.qsrinternational.com/nvivo-qualitative-data-analysis-software/home; 1999.

[80] Wetherell JL, Hershey T, Hickman S, Tate SR, Dixon D, Bower ES (2017). Mindfulness-based stress reduction for older adults with stress disorders and neurocognitive difficulties: a randomized controlled trial. J Clin Psychiatry.

[81] Nijjar PS, Puppala VK, Dickinson O, Duval S, Duprez D, Kreitzer MJ, et al. Modulation of the autonomic nervous system assessed through heart rate variability by a mindfulness based stress reduction program. Int J Cardiol. 2014;177(2) 10.1016/j.ijcard.2014.08.116 25179555

[82] Belleville S, Bherer L. Biomarkers of cognitive training effects in aging. Curr Transl Geriatr Exp Gerontol Rep. 2012; 10.1007/s13670-012-0014-5 PMC3693427 23864998

[83] Mirabito G, Verhaeghen P. The effects of mindfulness interventions on older adults’ cognition: A Meta-analysis. J Gerontol: Series B. 2022; 10.1093/geronb/gbac143 36148552

[84] Shahidi F, Ramraj C, Sod-Erdene O, Hildebrand V, Siddiqi A. The impact of social assistance programs on population health: A systematic review of research in high-income countries. BMC Public Health. 2019;19(1) 10.1186/s12889-018-6337-1 PMC6318923 30606263

[85] Fratiglioni L, Paillard-Borg S, Winblad B. An active and socially integrated lifestyle in late life might protect against dementia. Lancet Neurol. 2004;3 10.1016/S1474-4422(04)00767-7 15157849

[86] White P. Migrant populations approaching old age: prospects in Europe. J Ethn Migr Stud. 2006;32(8)

[87] World Medical Association declaration of Helsinki: Ethical principles for medical research involving human subjects. Vol. 310, JAMA. 2013. 10.1001/jama.2013.281053 24141714

